# Draft Genome Sequence of *Syntrophomonas wolfei* subsp. *methylbutyratica* Strain 4J5^T^ (JCM 14075), a Mesophilic Butyrate- and 2-Methylbutyrate-Degrading Syntroph

**DOI:** 10.1128/genomeA.00047-16

**Published:** 2016-03-03

**Authors:** Takashi Narihiro, Masaru K. Nobu, Hideyuki Tamaki, Yoichi Kamagata, Wen-Tso Liu

**Affiliations:** a National Institute of Advanced Industrial Science and Technology (AIST), Tsukuba, Ibaraki, Japan; b Department of Civil and Environmental Engineering, University of Illinois at Urbana-Champaign, Urbana, Illinois, USA

## Abstract

*Syntrophomonas wolfei* subsp. *methylbutyratica* strain 4J5^T^ (=JCM 14075^T^) is a mesophilic bacterium capable of degrading butyrate and 2-methylbutyrate through syntrophic cooperation with a partner methanogen. The draft genome sequence is 3.2 Mb, with a G+C content of 45.5%.

## GENOME ANNOUNCEMENT

*Syntrophomonas wolfei* subsp. *methylbutyratica* strain 4J5^T^ (=JCM 14075^T^) is one of a few isolated bacteria capable of volatile fatty acid (VFA) degradation through syntrophic association with a partner hydrogenotrophic methanogen (1–3). Strain 4J5^T^ was isolated from rice field mud in Jiangxi Province, China, and tentatively assigned to the genus *Syntrophomonas* (4). Known *Syntrophomonas* species all catabolize butyrate, and some also catabolize long-chain fatty acids in coculture with a partner methanogen (1) and play an important role in anaerobic ecosystems, such as sludge digestion (5, 6) and rice paddy fields (7). Of these *Syntrophomonas*-related syntrophs, strain 4J5^T^ is unique for its ability to degrade 2-methylbutyrate (4), suggesting that strain 4J5^T^ likely contributes to methanogenic degradation of organic compounds.

Strain JCM 14075^T^ was provided by the RIKEN BioResource Center (BRC) through the National Bio-Resource Project of the Ministry of Education, Culture, Sports, Science, and Technology (MEXT), Japan. Whole-genome shotgun sequencing was performed using the Illumina MiSeq platform (Illumina, San Diego, CA, USA) at the FASMAC Co., Ltd. (Atsugi, Japan). We constructed and sequenced a paired-end library (3,810,689 pairs; 2.26 Gb) and performed assembly using SPAdes (version 3.1.1) (8). The final assembly implemented 300 Mb of MiSeq paired-end reads (500,000 pairs), which provided 714.5× coverage of the draft genome. The JCM 14075^T^ draft genome is 3.2 Mb, contains a total of 89 scaffolds, and has a G+C content of 45.5%. Prokka (version 1.80) annotated 2,964 protein-coding genes, of which 2,186 have predicted functions, and 778 are hypothetical proteins (9). This pipeline also predicted 59 RNA genes, consisting of genes encoding 11 rRNAs (partial) and 43 tRNAs. We are currently exploring this genome to identify butyrate and 2-methylbutyrate degradation genes and pathways for further comparison with fatty-acid-degrading syntrophs.

### Nucleotide sequence accession numbers.

This draft genome sequence has been deposited at DDBJ/GenBank/EMBL under accession numbers BBQT01000001 to BBQT01000092.
